# Breast cancer risk in relation to occupations with exposure to carcinogens and endocrine disruptors: a Canadian case–control study

**DOI:** 10.1186/1476-069X-11-87

**Published:** 2012-11-19

**Authors:** James T Brophy, Margaret M Keith, Andrew Watterson, Robert Park, Michael Gilbertson, Eleanor Maticka-Tyndale, Matthias Beck, Hakam Abu-Zahra, Kenneth Schneider, Abraham Reinhartz, Robert DeMatteo, Isaac Luginaah

**Affiliations:** 1 Occupational and Environmental Health Research Group, Centre for Public Health and Population Health Research, University of Stirling, Stirling, Scotland, FK9 4LA, UK; 2 Department of Sociology, Anthropology, and Criminology, University of Windsor, 401 Sunset Avenue, Windsor, ON, N9B 3P4, Canada; 3 Education and Information Division, National Institute for Occupational Safety and Health (NIOSH), 4676 Columbia Parkway, Cincinnati, Ohio, 45226, USA; 4 Queen’s University Belfast, University Road, Belfast, Northern Ireland, BT7 1NN, UK; 5 Windsor Regional Cancer Centre, 2220 Kildare Road, Windsor, ON, N8W 2X3, Canada; 6 Occupational Health Clinics for Ontario Workers, 15 Gervais Drive, Suite 601, Don Mills, ON, M3C1Y8, Canada; 7 Department of Geography, Social Science Centre, University of Western Ontario, London, ON, N6A 5C2, Canada

**Keywords:** Agriculture, Breast cancer, Canning, Casino, Carcinogen, Endocrine disruptor, Metals, Occupational, Plastics

## Abstract

**Background:**

Endocrine disrupting chemicals and carcinogens, some of which may not yet have been classified as such, are present in many occupational environments and could increase breast cancer risk. Prior research has identified associations with breast cancer and work in agricultural and industrial settings. The purpose of this study was to further characterize possible links between breast cancer risk and occupation, particularly in farming and manufacturing, as well as to examine the impacts of early agricultural exposures, and exposure effects that are specific to the endocrine receptor status of tumours.

**Methods:**

1005 breast cancer cases referred by a regional cancer center and 1146 randomly-selected community controls provided detailed data including occupational and reproductive histories. All reported jobs were industry- and occupation-coded for the construction of cumulative exposure metrics representing likely exposure to carcinogens and endocrine disruptors. In a frequency-matched case–control design, exposure effects were estimated using conditional logistic regression.

**Results:**

Across all sectors, women in jobs with potentially high exposures to carcinogens and endocrine disruptors had elevated breast cancer risk (OR = 1.42; 95% CI, 1.18-1.73, for 10 years exposure duration). Specific sectors with elevated risk included: agriculture (OR = 1.36; 95% CI, 1.01-1.82); bars-gambling (OR = 2.28; 95% CI, 0.94-5.53); automotive plastics manufacturing (OR = 2.68; 95% CI, 1.47-4.88), food canning (OR = 2.35; 95% CI, 1.00-5.53), and metalworking (OR = 1.73; 95% CI, 1.02-2.92). Estrogen receptor status of tumors with elevated risk differed by occupational grouping. Premenopausal breast cancer risk was highest for automotive plastics (OR = 4.76; 95% CI, 1.58-14.4) and food canning (OR = 5.70; 95% CI, 1.03-31.5).

**Conclusions:**

These observations support hypotheses linking breast cancer risk and exposures likely to include carcinogens and endocrine disruptors, and demonstrate the value of detailed work histories in environmental and occupational epidemiology.

## Introduction

Breast cancer is the most frequent cancer diagnosis among women in industrialized countries and North American rates are amongst the highest in the world [1]. There is now evidence of associations with numerous lifestyle, genetic, physiological, and pharmaceutical risk factors [2], but these factors do not fully explain breast cancer etiology. There are likely multiple factors, some as yet unknown, that may be contributors [3]. While the association of breast cancer risk with specific avoidable environmental or occupational exposures remains unknown or contested [4,5], there is increasing understanding of the mechanistic complexity of the disease and the diversity of potential etiologic agents [6].

Lifetime exposures to endogenous estrogen affect the risk of breast cancer [7,8], and exogenous estrogenic compounds may do so as well [9,10]. Endocrine disruptor theory not only implies that the timing of exposure is important due to varying susceptibility, particularly during critical periods of breast development when breast tissue is less differentiated [11,12] but also predicts that effects may occur at low doses [13]. Rudel et al. identified 216 chemicals as mammary gland carcinogens in experimental animals [14], many of which have also been listed as potential endocrine disrupting chemicals (EDCs) [9]. These findings indicate an opportunity to evaluate these chemicals and the risk of breast cancer in occupationally exposed women [15].

Research regarding occupational exposures and breast cancer risk has generally been a neglected topic. Work-history based occupational breast cancer studies often lack demographic and reproductive status information [16-18]. Studies with adequate demographic and reproductive status information often lack detailed work history data beyond current employment [19,20]. There are three published studies of occupation and breast cancer with detailed work and reproductive histories similar to the present study [21-23].

This study was conducted in Essex and Kent counties of Southern Ontario, a region with a stable population and diverse modern agriculture and industry. A geographic clustering of excess breast cancer has persisted there over time [24]. In the early1990s staff at the Windsor Regional Cancer Centre (WRCC) and at the Occupational Health Clinic for Ontario Workers in the Essex-Kent region of Ontario, raised concerns about the numbers of industrial workers developing cancer [25]. Two exploratory breast cancer case–control studies were undertaken by a multidisciplinary team of occupational and environmental researchers but had limited statistical power and exposure assessment. The first was a hypothesis-generating multi-cancer case control study [26]; the second study focussed exclusively on breast cancer [27].

The prior hypotheses of the current breast cancer study were based on: a) previous work on the environmental causes of breast cancer, b) current theories of carcinogenesis and endocrine effects, and c) findings of a previous breast cancer study that observed: increased risks among women with an occupational history of farming (OR = 2.8; 95% CI, 1.6 - 4.8) and among those who subsequently worked in auto-related manufacturing (OR = 4.0; 95% CI, 1.7 - 9.9), or in health care (OR = 2.3; (95% CI, 1.1 - 4.6) [27]. In the same geographic area of Ontario, the present study includes: a much larger sample from a later and distinct time period; a more detailed classification of potential exposures; and a more extensive compilation of non-occupational risk factor information. The hypotheses focus on a) exposures during critical periods of reproductive status, b) risks in relation to hormone receptors, which are found on the tumor cell surface and bind estrogen or other endocrine-active agents, and c) interactions between prior agricultural work and subsequent employment.

## Methods

The WRCC, the area’s regional cancer treatment center, referred subjects to this population-based case–control study. Ethics approval for research on human subjects, which includes prior informed consent, was obtained from the research ethics committees at both the Windsor Regional Hospital and the University of Windsor.

### Data collection

The survey instrument was derived from previously developed questionnaires [28-30] with special attention to reproductive developmental stages. The questionnaire captured reproductive risk factors such as: parity, duration of lactation, menstrual and menopausal history, use of hormone replacement therapy and oral contraceptives, and family history. Demographic and lifestyle risk factors included: income, education, physical activity, weight and body mass index (BMI), alcohol use, smoking history, and residential history. Up to 12 jobs were recorded for each participant, i.e. periods of employment. These included start and end dates for each job as well as free-text descriptions of job activities which were used to inform coding of occupation, industry and exposure. Work history was available in time units of one year.

### Recruitment

Cases were recruited over a six year period from mid 2002 through mid 2008 with the following criteria: new diagnosis of histologically confirmed breast cancers (ICD-9 Code – 174) [31], excluding recurrences; current residence in Essex or Kent Counties; willingness and ability to participate in a one to two hour interview with adequate language facility. Upon receipt of informed consent, names, addresses and telephone numbers of cases were provided by WRCC staff. Information outlining the study was mailed to each of the referrals and follow-up telephone calls were made by research personnel to schedule interviews. To minimize selective recruitment bias, the information disclosed the goal of understanding the causes of cancer but did not identify a focus on occupation or environment. After informed consent was obtained, the patient’s date of diagnosis and tumor pathology regarding estrogen receptor (ER) or progesterone receptor (PR) status were accessed.

Community controls from the same geographic study area were recruited from 2003 through 2007. Randomly selected households were obtained through computer-generated telephone numbers and linkage to mailing addresses. The same study information which was sent to cases, which made no reference to occupational or environmental factors, was mailed to potential controls and followed-up with telephone calls. Eligibility requirements were the same as the cases, with the exception that only one person per household was allowed and could have no prior history of breast or ovarian cancer. Interviewers followed a scripted recruitment and interview plan. Cases and controls were compensated $20 for their time.

### Exposure classification

Each job was coded using the North American Industry Classification System (NAICS) [32] and National Occupational Classification (NOC) [33]. Within each job, multiple NAICS and NOC classifications were allowed. In order to characterize exposures in the subjects’ work activities, all unique NAICS and NOC combinations that occurred in the study’s collective work history were compiled and classified in one of 32 sectors, which we identified as “minor sectors” and in 8 sectors that we identified as “major” sectors of primary interest which were based on prior hypotheses and consideration of potential exposures to mammary carcinogens [14] or EDCs [34] (Table 1). Several minor sectors potentially of interest for breast cancer investigation, such as textiles, footwear, printing, ceramics, furniture, jewelry and electronics, were combined as light manufacturing due to small numbers of cases.

**Table 1:** Major and minor sectors, and counts of controls and cases by minor sector of longest duration

**Major sector**		**Minor sector**	**Controls**	**Cases**
			1146	1006
1	Farming	Agriculture/plants	23	37
		Agriculture/animals	3	5
2	Non-plastics light manufacturing	Textile manufacturing	3	5
		Footwear manufacturing	0	0
		Wood manufacturing	2	2
		Printing	8	6
		Electrical and electronics mfr	1	1
		Jewelry, furniture manufacturing	5	1
		Glass, ceramic manufacturing	2	1
3	Petroleum/Petrochemical	Petroleum, petrochemical, chemical manufacturing	8	6
4	Plastics	Plastics manufacturing (nonauto)	3	0
		Plastics manufacturing (auto)	9	26
5	Metal-related	Metallurgical, metalworking, metal fabrication	64	75
6	Transportation	Transportation	37	26
7	Cleaning/beauty care	Beauty salon/hair care	25	14
		Dry cleaning, laundry	2	8
8	Bars/gambling	Bars/gambling	11	16
	*Not categorized as “major”*	Mining	1	0
		Power Generation/distribution	4	5
		Construction	6	6
		Food manufacturing	10	30
		Liquor/beer/wine	12	6
		Tobacco manufacturing	1	1
		Media, culture	30	15
		Adm. non education or healthcare	242	229
		Education	176	149
		Healthcare	195	154
		Entertainment	13	5
		Hotels and motels	7	5
		Retail	193	124
		Restaurants, food services	46	36
		No employment reported	4	12

Sector duration lagged 5 yr (duration in sector until 5 yr prior to study survey).

Minor sectors based on mutually exclusive grouping of NAICS/NOC codes from all jobs reported.

Each unique NAICS/NOC combination was further assigned an exposure classification code signifying the likely presence and intensity of carcinogen and/or EDC exposures in the manner of expert panel assignment [28,35]. Investigators, who were blind to case/control status, assigned exposure categories as “low, moderate or high” based on general process characteristics and prior professional knowledge of chemical hazards. For example, Table 2 displays the NAICS/NOC combinations in the Plastics major sector and the assigned exposure codes. This assignment was implemented by investigators with extensive experience 1) in exposure assessment within the occupational health clinic network associated with the Ontario Workers Compensation system and 2) in a wide range of industrial hygiene evaluations including automobile and parts manufacturing, health care, casinos, food production and agriculture. The assigned exposure strata were randomly checked by team members to ensure consistency and validity. NAICS/NOC codes also determined a social class variable (white collar, blue collar, unknown) based on NOC text.

**Table 2:** Example of exposure category assignments; for Plastics, Major Sector 4

**NAICS**	**NOC**	**NAICS description**	**NOC description**	**Exposure**
326160	9214	Plastics Bottle Manufacturing	Supervisors, Plastic and Rubber Products Manufacturing	2
326191	9422	Plastics Plumbing Fixture Manufacturing	Plastics Processing Machine Operators	3
326191	9495	Plastics Plumbing Fixture Manufacturing	Plastic Products Assemblers, Finishers and Inspectors	3
326199	1411	All Other Plastics Product Manufacturing	General Office Clerks	1
326199	3152	All Other Plastics Product Manufacturing	Registered Nurses	2
326199	9422	All Other Plastics Product Manufacturing	Plastics Processing Machine Operators	3
326199	9495	All Other Plastics Product Manufacturing	Plastic Products Assemblers, Finishers and Inspectors	3
326199	9619	All Other Plastics Product Manufacturing	Other Labourers in Processing, Manufacturing and Utilities	3
326150	1411	Urethane and Other Foam (except Polystyrene)	General Office Clerks	1
326150	3152	Urethane and Other Foam (except Polystyrene)	Registered Nurses	2
326150	9482	Urethane and Other Foam (except Polystyrene)	Motor Vehicle Assemblers, Inspectors and Testers	3
326193	1411	Motor Vehicle Plastics Parts Manufacturing	General Office Clerks	1
326193	3152	Motor Vehicle Plastics Parts Manufacturing	Registered Nurses	2
326193	6641	Motor Vehicle Plastics Parts Manufacturing	Food Counter Attendants, Kitchen Helpers, Related Occup.	2
326193	9422	Motor Vehicle Plastics Parts Manufacturing	Plastics Processing Machine Operators	3
326193	9451	Motor Vehicle Plastics Parts Manufacturing	Sewing Machine Operators	3
326193	9482	Motor Vehicle Plastics Parts Manufacturing	Motor Vehicle Assemblers, Inspectors and Testers	3
326193	9495	Motor Vehicle Plastics Parts Manufacturing	Plastic Products Assemblers, Finishers and Inspectors	3
326193	9496	Motor Vehicle Plastics Parts Manufacturing	Painters and Coaters – Industrial	3
326193	9514	Motor Vehicle Plastics Parts Manufacturing	Metalworking Machine Operators	3
326193	9619	Motor Vehicle Plastics Parts Manufacturing	Other Labourers in Processing, Manufacturing and Utilities	3
326291	1411	Rubber Product Manufacturing for Mechanical Use	General Office Clerks	1
326291	2211	Rubber Product Manufacturing for Mechanical Use	Chemical Technologists and Technicians	2
326291	9495	Rubber Product Manufacturing for Mechanical Use	Plastic Products Assemblers, Finishers and Inspectors	3
326291	9615	Rubber Product Manufacturing for Mechanical Use	Labourers in Rubber and Plastic Products Manufacturing	3
326291	9616	Rubber Product Manufacturing for Mechanical Use	Labourers in Textile Processing	3
326291	9619	Rubber Product Manufacturing for Mechanical Use	Other Labourers in Processing, Manufacturing and Utilities	3
332813	9422	… Plating, Polishing, Anodizing, and Coloring	Plastics Processing Machine Operators	3
336320	9422	Motor Vehicle Electrical and Electronic Equip.	Plastics Processing Machine Operators	3
336360	9422	Motor Vehicle Seating and Interior Trim Mfr	Plastics Processing Machine Operators	3

Minor sectors: Plastics manufacturing (nonauto) and Plastics manufacturing (auto).

Exposure classification: low (1), moderate (2), and high (3).

### Exposure metrics

In preliminary analyses minor sectors were examined as: a) categorical variables (minor sector of longest [lagged] duration, a mutually exclusive classification); and b) continuous variables (lagged durations of employment in all minor sectors) [36]. Next, cumulative exposure metrics were calculated as the sum over time of the assigned exposure levels from each NAICS/NOC activity using two weighting schemes. The first assigned to the categories “low, moderate and high” the weights 0, 1 and 2, respectively, and summed these over time; the second assigned the weights 0, 1 and 10. The two weightings permitted a choice to be made concerning the ratio of average exposure levels in the “moderate” vs. “high” categories, which would be expected to vary widely across processes, workplaces and sectors. When a job comprised multiple NAICS/NOC categories (because of mixed activities, holding more than one position at a time, or sequential employment within one year) equal weight was assigned to each element of the job in assessing exposure. Duration and cumulative exposure metrics were lagged 5 years, i.e., summed up until 5 years prior to a subject’s diagnosis (cases) or participation in study (controls), accounting for delay between primary carcinogenic events and clinical diagnosis. Cumulative exposures were calculated: a) generically combining all sectors, b) within the eight major sectors, and c) for some additional groupings of special interest, some of which were derived from preliminary observations such as in food manufacturing or automotive plastics.

Because food canning was a major activity in this region, related exposures were examined. Polymer lining of cans was approved in the 1960’s by the US Food and Drug Administration [37] and became widespread, internationally, in the 1970’s. To test for a role of chemicals in canning, we defined canning exposure as work in the canning industry NAICS codes after 1973 by which time epoxy coatings were being widely introduced [38]. Food and beverage can coatings have been found to contain bisphenol A (BPA), which is a recognized EDC [39].

### Effect modification with prior employment in agriculture

Prior research suggested that early employment in agriculture may predispose individuals to higher risk from subsequent occupational exposures [27]. A biological interaction term for cumulative exposure and prior agricultural work was constructed for several of the major-sectors of concern (e.g., automotive plastics, canning) as a weighted sum over time of the sector exposures, where the weight was the then-current cumulative exposure in (prior) farm work. The exposure contribution from a given year was the exposure level rating of a job multiplied by the person’s cumulative, previous, agricultural exposure. Models were then fit with the usual cumulative exposure terms for major sectors together with these interaction terms.

### Critical time-windows

Cumulative exposures for some analyses were partitioned into time (age) windows representing distinct stages of breast development that could affect risk [12,40]. Cumulative exposure accruing in each window was calculated, with windows defined on age as follows: a) before menarche, b) menarche to first full term pregnancy, c) first full term pregnancy to menopause, d) after menopause. In the absence of a first completed pregnancy or premenopausal status, subjects would have no observation time in windows c or d, respectively.

### Statistical analysis

Results were based on frequency-matched case–control analyses using a loglinear specification in multiple conditional logistic regression [41]. Matching was achieved by stratifying the cases and controls in three-year age intervals such that, on average, ages of controls were within about 1.5 years of the cases. Due to sparse data, all subjects below age 30 were assigned the same matching stratum. Odds ratios (OR) from logistic regression models are interpreted as estimates of relative “risk” throughout this report. In addition to reproductive risk factors, demographic risk factors were included in all models, including: smoking (pack-years and pack-years squared) calculated up to the age of diagnosis/participation, education in three levels (less than high school, high school and some college, college degree), and family income (<$40,000, >$40,000 blue collar, >$40,000 white collar). Employment duration terms (linear and squared) were statistically significant and included in all matched analyses (except the initial descriptive analysis by minor sector of longest duration). For investigation of breast cancer restricted to specific receptor classifications, breast cancer cases of other types were excluded. Only three estrogen/progesterone receptor categories were examined due to small numbers of cases in other types: ER+/PR+; ER+/PR-; ER-. For examination of menopausal status, subjects were classified on whether age was greater than age at menopause when augmented with a five-year lag. Additive relative rate model specifications were also evaluated using conditional logistic regression [42], which permitted testing for effect measure modification, or interaction, in an additive model context.

The results display both p-values, showing the probabilities of chance associations, and confidence intervals, showing the range of true parameter values that would produce the observed estimates with probability > 2.5 percent (two-tailed).

## Results

Of 1,553 breast cancer cases referred, 160 were ineligible and 222 were unable to be reached. Of the remainder, 165 declined, leaving 1006 cases for a participation rate of 86%. Of 3,662 households contacted for community control recruitment, 3,223 individuals were able to be apprised of the study and 926 households (29%) were determined to have no eligible residents. From 2,297 households with eligible residents, 1,146 women participated for a recruitment rate of 50%. The same percentages of cases and controls elected to be interviewed by telephone (46%) and in-person (54%).

Compared to controls, cases were slightly older, had a longer period of fecundity (from menarche to menopause or participation date, whichever came earlier) and fewer months of breast feeding; they had less education, lower family income, and smoked more but had almost identical duration of employment (Table 3). There is no information available regarding the occupational histories of non-participants or expected employment sector distribution. However, it is unlikely, based on the almost identical duration of employment of cases and controls, that employment status influenced participation. Moreover, during recruitment, the research focus on occupation was not known to potential participants and therefore would not have biased participation. The differences between cases and controls, which were potentially confounding, were adjusted for in the age-matched statistical models. The difference in average date of participation (controls) vs. average date of diagnosis (cases), which determined when exposure assessment ceased, was less than 6 months (Table 3).

**Table 3:** Descriptive statistics for breast cancer cases and controls

	**Controls**	**Cases**
n	1146	1006
Age @ interview, years, mean	56.2	60.0
Year @ interview (controls), or diagnosis (cases), mean	2006.3	2005.8
Never pregnant, %	11.9	11.9
Number of full-term pregnancies, mean	2.83	2.84
Duration fecundity, year, mean	32.2	33.9
Total breastfeeding, mo, mean	5.8	4.9
Education < HS, %	13.3	23.6
Education = HS or some college, %	40.1	38.7
Education > HS and some college, %	46.6	37.7
Family annual income < $40,000, %	31.3	46.8
Family income >= $40,000 and bluecollar, %	22.5	17.5
Family income >= $40,000 and whitecollar, %	46.2	35.7
Pack-years of smoking (lagged 5 year), mean	6.39	7.52
Duration employed (lagged 5 year), year, mean	25.7	25.5
**Cumulative exposure in Major Sectors**^**1**^		
Farming, mean	7.19	12.06
Non-plastic light mfg, mean	1.21	1.39
Petrochemical, mean	0.12	0.12
Plastics mfg, mean	1.99	4.13
Metalworking, mean	2.33	4.50
Transportation, mean	0.88	0.71
Beauty care, laundry/dry cleaning, mean	0.39	0.39
Bars-gambling, mean	0.11	0.17

1 cumulative exposure on transformed ratings: 1 (low), 2 (moderate), 3 (high) → 0, 1, 10, as rating-year.

There were considerably more cases than controls among subjects whose minor sector of longest duration was a) agriculture: 37 vs. 23 cases, b) food manufacturing: 30 vs. 10, c) automotive plastics manufacturing: 26 vs. 9, d) laundry/dry cleaning: 8 vs. 2 and e) bars-gambling (16 vs. 11) (Table 1). Very few subjects reported no employment (4 controls, 8 cases; Table 1). Cumulative exposure was similar or less in cases versus controls in some major sectors of interest – petrochemicals, transport, beauty care/laundry/dry cleaning – but considerably higher in farming, plastics manufacturing, metallurgical/metalworking and bars-gambling work.

When classified on minor sector of longest (lagged) duration of employment, and analyzed with conditional logistic regression, several demographic and reproductive risk factors exhibited strong, statistically significant associations as did several minor sectors of employment (Table 4). The odds of being a breast cancer case were 5 percent lower with each additional pregnancy, and greater by 2.5 percent for each additional year of fecundity. The odds were 47 percent higher for women with less than high-school education. The odds, with a family income higher than $40,000, were lower for both blue collar workers (43 percent lower) and white collar workers (37 percent lower). Risk of breast cancer was higher per pack-year in smokers (OR = 1.02; 95% CI, 1.00-1.04) but with a slight attenuation of effect with increasing pack-years (negative quadratic term). For 20 pack-years, the smoking OR was exp(20×0.019-400×0.00033) = 1.28. The minor employment sectors showing elevated odds of breast cancer were food manufacturing (OR = 2.25; 95% CI, 0.97-5.26) and automotive plastics manufacturing (OR = 3.12; 95% CI, 1.29-7.55). Both laundry/dry cleaning and bars-gambling work were associated with increased odds of breast cancer (OR= 2.72, 95% CI, 0.56-13.2 and OR = 1.79, 95% CI, 0.73-4.41, respectively) that were not statistically significant because of small numbers. In this model, work in any other sector than the longest was disregarded. The restaurant sector was the reference group in this analysis (with a mutually exclusive and exhaustive classification, one sector must play that role). Analyses were repeated specifying the large retail sector as reference (data not shown). That sector appeared to have less than average breast cancer risk (Tables 1, 3) and, as a result, all the estimates for other sectors increased considerably when compared to retail. For example, the automotive plastics OR increased from 3.12 to 5.38 (95% CI, 2.34-12.4).

**Table 4:** Matched case–control analysis for breast cancer incidence with classification on minor sector of longest duration, and reproductive and demographic risk factors: full model, by conditional logistic regression

**Parameter**	**Estimate**	**Chi-Sq**	**Wald P**	**OR (95% CI)**
Ind: never pregnant	−0.078	0.23	0.64	0.93 (0.67-1.28)
Number of full-term pregnancies	−0.054	4.05	0.044	0.95 (0.90-1.00)
Duration fecundity, year	0.025	14.94	0.0001	1.03 (1.01-1.04)
Total breastfeeding, mo	−0.004	0.93	0.33	1.00 (0.99-1.00)
Ind: education < high-school	0.387	7.34	0.0067	1.47 (1.11-1.95)
Ind: education > high-school and some college	−0.099	0.81	0.37	0.91 (0.73-1.12)
Ind: family income >= $40,000 and bluecollar	−0.559	15.98	<.0001	0.57 (0.44-0.75)
Ind: family income >= $40,000 and whitecollar	−0.464	15.90	<.0001	0.63 (0.50-0.79)
Pack-years of smoking (lagged 5 year)	0.019	4.54	0.033	1.02 (1.00-1.04)
Pack-years of smoking, squared	−3.3 10^-4^	3.00	0.083	1.00 (1.00-1.00)
**Minor sector of longest duration (lagged 5 year)**				
Agriculture/plants	0.219	0.39	0.53	1.25 (0.63-2.47)
Agriculture/animals	0.696	0.79	0.37	2.01 (0.43-9.28)
Mining	−1.782	0.30	0.59	0.17 (0.00-102.)
Power Generation/distribution	0.435	0.37	0.54	1.55 (0.38-6.31)
Construction	0.245	0.15	0.70	1.28 (0.37-4.46)
Food manufacturing	0.812	3.53	0.060	2.25 (0.97-5.26)
Liquor/beer/wine	−0.849	2.29	0.13	0.43 (0.14-1.29)
Tobacco manufacturing	−0.984	0.44	0.51	0.37 (0.02-6.83)
Textile manufacturing	0.549	0.49	0.48	1.73 (0.37-8.04)
Wood manufacturing	−0.109	0.01	0.92	0.90 (0.11-7.05)
Printing	−0.307	0.26	0.61	0.74 (0.23-2.40)
Petroleum, petrochemical, chemical mfr	−0.294	0.24	0.63	0.75 (0.23-2.43)
Plastics manufacturing (non-auto)	−3.211	0.75	0.39	0.04 (0.00-58.0)
Plastics manufacturing (auto)	1.137	6.34	0.012	3.12 (1.29-7.55)
Glass, ceramic manufacturing	−0.895	0.47	0.49	0.41 (0.03-5.24)
Metallurgical, metalworking and fabrication	0.118	0.18	0.67	1.13 (0.65-1.94)
Electrical and electronics manufacturing	−0.357	0.06	0.81	0.70 (0.04-12.3)
Jewelry, furniture manufacturing	−2.141	2.86	0.091	0.12 (0.01-1.41)
Retail	−0.470	3.60	0.058	0.63 (0.39-1.02)
Transportation	−0.258	0.58	0.45	0.77 (0.40-1.50)
Media, culture	−0.688	3.05	0.081	0.50 (0.23-1.09)
Administration (non educ, non healthcare)	0.000	0.00	0.99	1.00 (0.62-1.61)
Education	0.032	0.02	0.90	1.03 (0.62-1.71)
Healthcare	−0.104	0.18	0.67	0.90 (0.56-1.46)
Entertainment	−0.943	2.51	0.11	0.39 (0.12-1.25)
Hotels and motels	−0.090	0.02	0.89	0.91 (0.26-3.19)
Beauty salon/hair care	−0.491	1.45	0.23	0.61 (0.28-1.36)
Drycleaning, laundry	1.000	1.54	0.21	2.72 (0.56-13.2)
Bars, gaming/gambling	0.582	1.59	0.21	1.79 (0.73-4.41)

OR – odds ratio, 95% CI – 95% confidence interval, Ind – (0,1) indicator variable.

Matching on age in 3 year- intervals.

Reference category: minor sector = Restaurants, food services / age = 40 / Education = high-school or some college / blue collar / Family annual income < $40,000 / Ever-pregnant, zero births / non smoker.

When durations in the minor sectors (lagged) were analyzed in the model (Table 5), food manufacturing and dry cleaning/laundry were no longer elevated, but agriculture/plants minor sector was elevated (OR=1.02 per year, 95%CI=0.99-1.05), and plastics manufacturing (auto) (OR=1.09 per year, 95%CI=1.03-1.15; p=0.0023) now had a more significant effect (χ2=9.25 vs. 6.97), implying an improved model fit. One year in plastics (auto) employment was estimated to increase the odds of breast cancer by 9 percent. Inclusion of terms for total employment duration (lifetime employment as of study age) and the square of that term, produced a better fitting model with breast cancer risk declining with total employment (χ2(2df) =5.84, p=0.05).

**Table 5:** Breast cancer odds ratios (matched analysis) for duration (lagged) in minor sectors excluding terms for sectors likely to have low work-related risk (mass media, education, healthcare, entertainment)

**Parameter**	**OR (95% CI)**	**Wald P**
**Duration in minor sectors, year (lagged 5 year)**		
Agriculture/plants	1.02 (0.99-1.05)	0.14
Agriculture/animals	1.02 (0.96-1.08)	0.54
Mining	0.82 (0.53-1.29)	0.39
Power Generation/distribution	1.02 (0.96-1.08)	0.59
Construction	1.01 (0.94-1.08)	0.84
Food manufacturing	1.02 (0.99-1.06)	0.24
Liquor/beer/wine	0.99 (0.95-1.03)	0.50
Tobacco manufacturing	0.91 (0.77-1.09)	0.30
Textile manufacturing	1.06 (0.97-1.16)	0.21
Wood manufacturing	0.77 (0.58-1.03)	0.075
Printing	1.05 (0.96-1.15)	0.27
Petroleum, petrochemical, chemical mfr	0.98 (0.93-1.03)	0.42
Plastics manufacturing (non auto)	0.86 (0.69-1.06)	0.16
Plastics manufacturing (auto)	1.09 (1.03-1.15)	0.0023
Glass, ceramic manufacturing	1.01 (0.91-1.12)	0.89
Metallurgical, metalworking and fabrication	1.01 (0.99-1.03)	0.25
Electrical and electronics manufacturing	1.03 (0.93-1.13)	0.61
Light manufacturing (jewelry, furniture	0.96 (0.84-1.09)	0.52
Retail	0.98 (0.97-1.00)	0.012
Transportation	0.98 (0.96-1.01)	0.29
Hotels and motels	0.96 (0.89-1.03)	0.23
Beauty salon/hair care	0.99 (0.95-1.02)	0.50
Drycleaning, laundry	1.02 (0.95-1.09)	0.64
Bars, gaming/gambling	1.00 (0.96-1.05)	0.91
Restaurants, food services	1.01 (0.98-1.03)	0.68
**Total employment duration, year (lagged 5 year)**^**1**^		
Duration	0.97 (0.93-1.00)	0.063
Duration, squared	1.00 (1.00-1.00)	0.18

Excluded minor sectors: Media, culture; Administration: non educ., non healthcare; Education; Healthcare; Entertainment.

Odds ratios (OR) from single model by conditional logistic regression with terms for demographic, reproductive risk factors as in Table 4 and terms for employment duration; matching on age in 3-year intervals.

OR evaluated at duration = 1year (lagged 5 year).

1. for including employment duration terms: χ2 (2df) =5.84, p=0.05.

### Models with cumulative exposure

Using the generic cumulative exposure metric (across all minor sectors) with the 0, 1, 2 exposure weighting scheme produced a statistically significant excess risk of breast cancer; 10 years in a high-exposed job had an associated 29% increase (OR = 1.29; 95% CI, 1.10-1.51) (Table 6, model 1). With the (0,1,10) weighting scheme, a stronger association resulted (OR = 1.42; 95% CI, 1.18-1.73), with a 42% increase in risk after 10 years in jobs assessed as likely high-exposure (model 2). Applying the 0, 1, 10 weighting scheme within major sectors identified excess breast cancer risk: in agriculture (OR = 1.34; 95% CI, 1.03-1.74; for 10 years in high-exposure jobs), plastics (OR = 2.43; 95% CI, 1.39-4.22), metal work (OR = 1.73; 95% CI, 1.02-2.92) and in bars-gambling work (OR = 2.20; 95% CI, 0.91-5.29) (model 3). There was no additional risk, beyond that found in farming in general, for specific farming activities involving corn cultivation since 1978 when atrazine use became common or greenhouse work. The excess in chemicals/petrochemicals was based on only 6 cases. Including additional terms for categories of special interest slightly strengthened the major category associations (Table 6, model 4).

**Table 6:** Breast cancer odds ratios (matched analysis) with cumulative exposures, in major sectors and for derived hypotheses, and interactions with prior agricultural work, by conditional logistic regression

**Model/Parameter**	**OR (95% CI)**	**Wald P**
**Model 1**		
Cumulative Exposure^1^ I (lagged 5 year)	1.29 (1.10-1.51)	0.0017
**Model 2**		
Cumulative Exposure^2^ II (lagged 5 year)	1.42 (1.18-1.73)	0.0003
**Model 3**		
Farming	1.34 (1.03-1.74)	0.031
Non-plastic light mfg	0.83 (0.29-2.37)	0.73
Chemical, petrochemical	2.15 (0.0->100)	0.82
Plastics	2.43 (1.39-4.22)	0.0018
Metalworking	1.73 (1.02-2.92)	0.041
Transport	0.84 (0.28-2.52)	0.76
Beauty care/laundry/dry cleaning	1.02 (0.72-1.43)	0.92
Bars/gambling	2.20 (0.91-5.29)	0.078
**Model 4**		
Farming: all	1.36 (1.01-1.82)	0.044
Farming: corn (since 1978)	0.76 (0.09-6.69)	0.80
Farming: greenhouse workers	1.04 (0.38-2.83)	0.94
Non-plastic light mfg	0.87 (0.30-2.50)	0.80
Chemical, petrochemical	1.47 (0.0->100)	0.91
Transport	0.80 (0.25-2.54)	0.71
Beauty care/laundry/dry cleaning	1.02 (0.72-1.43)	0.92
Bars/gambling	2.28 (0.94-5.53)	0.068
Auto industry: plastics	2.68 (1.47-4.88)	0.0013
Auto industry: small enterprises	2.48 (1.00-6.10)	0.051
Auto industry: large enterprises	1.18 (0.56-2.50)	0.66
Canning	2.35 (1.00-5.53)	0.050
Healthcare workers	1.01 (0.87-1.18)	0.89
Toll booth workers	1.17 (0.44-3.14)	0.76
**Model 5**		
Farming: all	1.35 (1.00-1.82)	0.049
Farming: corn (since 1978)	0.64 (0.07-5.78)	0.69
Farming: greenhouse workers	0.95 (0.35-2.60)	0.92
Non-plastic light mfg	0.86 (0.30-2.49)	0.78
Chemical, petrochemical	1.56 (0.0->100)	0.90
Metalworking	1.71 (0.99-2.95)	0.055
Metalworkingl… IpAg	1.04 (0.89-1.21)	0.64
Transport	0.82 (0.27-2.55)	0.74
Beauty care/laundry/dry cleaning	1.03 (0.73-1.45)	0.87
Bars, gambling	1.79 (0.67-4.73)	0.24
Bars, gambling … IpAg	2.38 (0.58-9.79)	0.23
Auto industry: plastics	2.41 (1.31-4.44)	0.0048
Auto plastics… IpAg	2.31 (0.53-9.98)	0.26
Canning	1.90 (0.72-4.99)	0.19
Canning … IpAg	1.14 (0.83-1.56)	0.43
Healthcare workers	1.05 (0.89-1.24)	0.54
Healthcare… IpAg	0.96 (0.91-1.02)	0.20
Toll booth workers	1.17 (0.43-3.13)	0.76

All five models include reproductive, demographic risk factors as in Table 4 and employment duration terms; IpAg, interaction with farming: cumulative (sector rating × prior cum. exposure in agriculture).

Odds ratios (OR) evaluated at 10 years in high-exposed jobs (lagged 5 year) or, for interactions, at 10 years in high-exposed jobs and 1 year in prior high-exposed farm work; matching on age in 3-year intervals; for including employment duration terms: χ^2^ (2df) =10.9, p=0.025 (Model 4).

1. cumulative exposure on transformed ratings: 1 (low), 2 (moderate), 3 (high) → 0, 1, 2, as rating-year.

2. cumulative exposure on transformed ratings: 1 (low), 2 (moderate), 3 (high) → 0, 1, 10, as rating-year, except bars/gambling and toll booth workers (maximum rating = 1; no jobs rated high).

The analysis revealed excess risk with work in high exposure food canning jobs (OR = 2.35; 95% CI, 1.00-5.53, for 10 years work) (Table 6, model 4). This metric was motivated by the endocrine disruptor hypothesis and by preliminary findings of an excess in those for whom food manufacturing was the sector of longest duration (Table 4). There was a possible excess in a group that includes toll booth operators (with potentially high vehicle emission exposures) (OR = 1.17; 95% CI, 0.44-3.14) but this group was limited by small numbers. The strongest association was with automotive plastics manufacturing (OR = 2.68; 95% CI, 1.47-4.88, p=0.0013). Within the auto industry in general, excess breast cancer appeared to be limited to small automotive parts suppliers, which would include some plastics operations (OR = 2.48; 95% CI, 1.00-6.10).

### Effect modification and windows of vulnerability

There was no evidence of risk modification related to prior work in agriculture for subsequent work in metals or canning (Table 6, model 5). For bars-gambling work the estimate for the interaction term was stronger (OR=2.38, 95%CI=0.58-9.79; for 1 year of farm work prior to 10 years of bars-gambling exposure) than for the main effect, although both were not statistically significant (Table 6, model 5). For automotive plastics the estimate of a doubling of risk for one year of prior farm work was not statistically significant (OR=2.31, 95%CI=0.53-9.98).

Partitioning the generic Cumulative Exposure Metrics I and II into time-windows suggests that the most important exposures affecting breast cancer risk occur in the third time window – from first full term pregnancy to menopause; the elevation was smaller for the first, second and fourth time-windows although there was limited power to distinguish them (Table 7). For Metric II, the point estimates for the second and third windows were close. Exposures in farming and bars-gambling work exhibited the same pattern whereas for the metal-related, plastics, and canning metrics the most important period appeared to be the second time-window – menarche to first full term pregnancy – before breast tissue is fully differentiated.

**Table 7:** Breast cancer odds ratios for cumulative exposure accruing in time-windows reflecting reproductive status, by conditional multiple logistic regression

**Parameter**		
**Window**	**OR (95% CI)**	**Wald P**
**Cumulative Exposure**^**1**^**I**		
< menarche	1.037 (0.89-1.21)	
menarche-first pregnancy	1.018 (0.98-1.06)	
first pregnancy – menopause	1.036 (1.01-1.06)	0.012
menopause -	1.012 (0.97-1.06)	
**Cumulative Exposure**^**2**^**II**		
< menarche	1.003 (0.85-1.18)	
menarche-first pregnancy	1.037 (0.98-1.09)	0.18
first pregnancy – menopause	1.050 (1.01-1.09)	0.0072
menopause -	1.018 (0.97-1.07)	
**Selected cumulative exposures**^**2,3,4**^		
Farming		
< menarche	1.054 (0.88-1.26)	
menarche-first pregnancy	0.997 (0.93-1.07)	
first pregnancy – menopause	1.046 (0.98-1.12)	0.19
menopause -	1.054 (0.96-1.16)	
Bars, gambling		
menarche-first pregnancy	1.022 (0.81-1.29)	
first pregnancy – menopause	1.141 (0.98-1.33)	0.092
menopause -	1.039 (0.82-1.32)	
Metalworking		
menarche-first pregnancy	1.161 (0.96-1.40)	0.12
first pregnancy – menopause	1.064 (0.97-1.16)	0.17
menopause -	1.020 (0.92-1.13)	
Auto industry: plastics		
menarche-first pregnancy	1.297 (1.05-1.61)	0.018
first pregnancy – menopause	1.104 (1.01-1.20)	0.023
menopause -	1.044 (0.92-1.19)	
Canning		
menarche-first pregnancy	1.262 (0.96-1.66)	0.095
first pregnancy – menopause	1.079 (0.96-1.22)	
menopause -	1.041 (0.88-1.24)	

All three models include reproductive, demographic risk factors as in Table 4 and employment duration; matching on age in 3-year intervals.

OR for cumulative exposure evaluated at 1.0 year in time-window in high-exposed jobs (lagged 5 year).

1. cumulative exposure on transformed ratings: 1 (low), 2 (moderate), 3 (high) → 0, 1, 2, as rating-year.

2. cumulative exposure on transformed ratings: 1 (low), 2 (moderate), 3 (high) → 0, 1, 10, as rating-year.

3. model includes all major sector exposures;

4. no cases/controls with non-farm exposure in window: < menarche.

### Hormone receptor type and menopausal status

Examination of specific estrogen receptor (ER) or progesterone receptor (PR) types in the major sectors showing excess breast cancer produced distinct associations across receptor types (Table 8). The farming, metals, bars-gambling and particularly automotive plastics (OR = 3.63; 95% CI, 1.90-6.94, p=10^-4^) sectors all exhibited excesses for the ER+/PR+ receptor type, but farming had a stronger excess in the ER- category (OR = 1.71; 95% CI, 1.12-2.62, p=0.014). The canning excess appeared to be entirely in the ER+ /PR- and ER- groups. Including the interaction terms for prior farm work identified possible effect modification for metals (ER+/PR-), bars-gambling (ER+ /PR+), and plastics (ER-), and a statistically significant interaction for prior farming and canning for the ER+ /PR- receptor status (OR = 1.81; 95% CI, 1.08-3.04, p=0.025) but not for ER+/PR+ or ER- receptor status.

**Table 8:** Breast cancer odds ratios (matched analysis) in selected major sectors on tumor estrogen receptor status, and with interaction on prior farm work

**Tumor receptor status**	**ER+/PR+**	**ER+/PR-**	**ER-**
N cases (total=1006)	538	157	188
**Model 1 – Cumulative exposure,**^**1**^**no interactions**			
	OR (95% CI) Wald P (two-tailed)		
Farming	1.32 (0.94-1.85) 0.12	1.35 (0.73-2.49)	1.71 (1.12-2.62) 0.014
Metalworking	2.03 (1.11-3.71) 0.022	1.73 (0.77-3.89)	1.02 (0.36-2.89)
Bars, gambling	3.87 (1.39-10.8) 0.010	3.24 (0.44-24.1)	0.15 (0.00-4.27)
Auto industry: plastics	3.63 (1.90-6.94) 9×10^-5^	1.17 (0.28-4.97)	1.76 (0.78-3.94)
Canning	1.50 (0.55-4.10)	4.01 (1.37-11.8) 0.011	3.19 (1.16-8.75) 0.024
**Model 2 – Cumulative exposure with prior farm interaction terms (IpAg)**			
	OR (95% CI) Wald P (two-tailed)		
Farming	1.32 (0.93-1.87)	1.34 (0.70-2.57)	1.76 (1.13-2.74) 0.012
Metalworking	2.21 (1.14-4.30) 0.019	1.51 (0.65-3.50)	1.17 (0.43-3.13)
Metalworking … IpAg	0.84 (0.53-1.32)	1.26 (0.95-1.67) 0.11	0.47 (0.12-1.93)
Bars, gambling	2.87 (0.93-8.84) 0.066	2.78 (0.35-22.1)	0.20 (0.01-5.45)
Bars, gambling … IpAg	3.03 (0.74-12.4) 0.12	3.46 (0.27-45.0)	0.00 (0.00->100)
Auto industry: plastics	3.13 (1.62-6.05) 7×10^-4^	1.26 (0.30-5.32)	0.96 (0.31-2.99)
Auto plastics… IpAg	2.10 (0.52-8.43)	0.65 (0.03-15.3)	3.03 (0.80-11.6) 0.10
Canning	1.52 (0.51-4.51)	1.21 (0.26-5.60)	4.85 (1.25-18.8) 0.022
Canning… IpAg	0.91 (0.51-1.65)	1.81 (1.08-3.04) 0.025	0.62 (0.22-1.72)

Odds ratios (OR) by conditional logistic regression with terms for reproductive, demographic risk factors as in Table 4 and terms for employment duration; matching on age in 3-year intervals; models include all major sector exposures; IpAg, interaction with farming: cumulative (sector rating × prior cum. exposure in agriculture); breast cancer cases not of the specified receptor type were excluded from analysis.

OR for cumulative exposure evaluated at 10.0 year in high-exposed jobs (lagged 5 year) or, for interactions, at 10 years in high-exposed jobs and 1 year in prior high-exposed farm work.

Models fit with an additive relative rate specification generally fit less well than with the loglinear form. For example, the automotive plastics estimate with the loglinear model was OR=2.68 (1.47-4.88), p=0.0013 whereas the linear relative rate model produced OR=4.03 (1.43-6.64), p=0.023. With the interaction terms, the same pattern was observed as with the loglinear form, but confidence intervals were wider.

Restricting the analysis to premenopausal women resulted in many fewer cases (373 out of 1006) and considerably higher estimates of relative risk (Table 9) as in high exposed jobs in automotive plastics (OR=5.10, 95% CI=1.68-15.5) or canning (OR=5.20, 95% CI=0.95-28.4). Thus 10 yrs in that work was associated with a five-fold excess in breast cancer incidence. Adding a term for body mass index (BMI, centered at 25) produced a reduced odds of breast cancer with BMI (for 10 unit increase, OR = 0.78; 95% CI, 0.61-0.99), a slightly weaker association for automotive plastics, and a stronger association for canning (OR = 5.70; 95% CI, 1.03-31.5). In the analysis of postmenopausal breast cancer (633 cases), estimated risks associated with specific sectors were lower, particularly for automotive plastics and canning sectors. Terms for total employment duration, which were not statistically significant for premenopausal breast cancer, were statistically significant for postmenopausal cancer, with an estimated 6% decline in risk for each additional year of employment. BMI was a strong risk factor for postmenopausal breast cancer (for 10 unit increase in BMI, OR = 1.37; 95% CI, 1.12-1.68) but with small changes in major-sector risk estimates on addition of the BMI term.

**Table 9:** Breast cancer odds ratios (matched analysis) and menopausal status with BMI and selected risk factors and major sectors, by conditional logistic regression

**Model/ Parameter**	**OR (95% CI)**	**OR (95% CI)**	**OR (95% CI)**	**OR (95% CI)**
	**Wald P**	**Wald P**	**Wald P**	**Wald P**
	Premenopausal (373 cases)		Postmenopausal (633 cases)	
		BMI		BMI
Body Mass Index	-	0.78 ( 0.61-0.99) 0.048	-	1.37 (1.12-1.68) 0.0023
Smoking, pk-yrs	1.04 (1.00-1.08) 0.030	1.04 (1.00-1.08) 0.028	1.01 (0.99-1.03)	1.01 (0.99-1.03)
Employ. duration	0.98 (0.91-1.06)	0.99 (0.91-1.07)	0.94 (0.90-0.98) 0.0050	0.94 (0.90-0.98) 0.0046
Farming	1.64 (0.78-3.46)	1.62 (0.76-3.44)	1.34 (0.97-1.85) 0.079	1.35 (0.97-1.87) 0.073
Metalworking	1.72 (0.57-5.22)	1.57 (0.51-4.82)	1.84 (0.97-3.49) 0.061	1.83 (0.96-3.46) 0.065
Bars, gambling	2.32 (0.40-13.5)	2.55 (0.44-14.7)	2.05 (0.74-5.66)	2.15 (0.76-6.06)
Auto plastics	5.10 (1.68-15.5) 0.004	4.76 (1.58-14.4) 0.006	2.29 (1.12-4.67) 0.023	2.25 (1.09-4.66) 0.028
Canning	5.20 (0.95-28.4) 0.056	5.70 (1.03-31.5) 0.046	1.62 (0.63-4.17)	1.47 (0.55-3.97)

Definition of pre/postmenopausal population: age at diagnosis (cases) or survey (controls) was less than /greater or equal to (age at menopause plus 5 year lag).

Odds ratios (OR) by conditional logistic regression in single models with terms for reproductive, demographic risk factors as in Table 4 and terms for employment duration, cumulative exposures in all major sectors (lagged 5 year) and for Pack-years of smoking, squared; P – p-value, two tailed.

OR for cumulative exposure evaluated at 10.0 year in high-exposed jobs (lagged 5 year), for a BMI increase from 25 to 35.

## Discussion

Our objective was to identify occupations associated with elevated rates of breast cancer. This issue has been largely neglected, possibly because of class [43] and gender bias [44]. Many of the findings in this study are consistent with those from other studies of non-occupational risk factors for breast cancer related to the lifetime load of endogenous estrogens [6]. These include the finding of an increased risk with duration of fecundity, decrease with the number of pregnancies and a not statistically significant decrease with length of breast-feeding [45]. Similarly, the 28% increase associated with a 20 pack-year smoking history is consistent with other studies [46] indicating a general validity of the approach and findings. The observed socioeconomic status (SES) effect is less consistent with prior work. Although higher income and education have generally been associated with higher risk [47], our findings of an elevated risk in women with lower SES may have resulted from higher exposures to EDCs and carcinogens in the lower income manufacturing and agricultural industries of the geographic study area.

Band et al. [21] conducted a case–control study with 1018 cases in British Columbia. It was similar to this study but with separate analyses for large numbers of industry and occupation categories, classified as “usual” (longest held job) or ”ever/never,” and compared to all others. Because long durations of employment in one sector would tend to be associated with short durations in all other sectors, sectors conferring risk would be mutually negatively confounding when analyzed one at a time (i.e., the comparison group would have higher durations in the competing etiologic sectors). Beauty care, transportation, data-processing and food processing showed significant elevations for premenopausal breast cancer and laundry/drycleaning for postmenopausal cancer.

Villeneuve et al. [22] analyzed a case–control study (1230 cases) in two departments of France for each of 41 industry and 54 occupation categories individually, observing a statistically significant breast cancer excess after 10 years duration in motor vehicle manufacturing (obs/exp= 18/7=2.6(95% CI:1.0-6.3). This study also may have had negative exposure confounding causing diminished effect estimates.

Labrèche et al. [23] analyzed a case–control study in Montreal (556 cases) using an expert panel to estimate historical exposures to 300 substances. Analyses, limited to only 22 substances with > 5% prevalence, found significant excesses of postmenopausal cancer for polycyclic aromatic hydrocarbons (PAHs), and several polymeric fibers; results for chemicals involved in automotive plastics or canning operations were not reported.

Observing associations between breast cancer incidence and generically assigned exposure ratings in broad industrial categories in the present study suggests that the etiologic agents, whether as carcinogens or EDCs, are widely distributed possibly encompassing many compounds. The specific identification of causative agents should be possible in occupational studies with detailed compound-specific retrospective exposure assessments.

### Occupational sectors

Many of the women in this study had a background in farming or in the automotive plastics sector and this provided sufficient statistical power to show consistency with our prior studies [26,27]. Similarly, statistical power was sufficient to reliably identify elevated risks associated with food canning, bars-gambling and metalwork. In other sectors, such as construction, petrochemical, printing, and textile manufacturing sectors, there was a lack of statistical power.

### Farming

This study found elevated breast cancer risk among women who had farmed. Agriculture in southwestern Ontario is diverse with tomato, corn and peach production being major activities. No additional risk, beyond what was found for farming in general, was observed for corn cultivation when atrazine was used but the labor-intensive activities there (detasseling) may have had low exposures. Several pesticides act as mammary carcinogens in animal bioassays [14]; many are EDCs [40]. In several cohort studies no elevated risk was observed among farming women [4] but some of these studies did not examine specific exposures or their timing. The Agricultural Health Study [48], while inconclusive, found risk was elevated among postmenopausal women whose husbands used specific pesticides [49]. A recent study found that young women exposed to DDT before the age of fourteen had an excess breast cancer risk before age fifty [50]. Band et al. [21] found in pre- and postmenopausal cases (combined) elevated breast cancer risk in fruit and other vegetable farming (OR = 3.11, 90% CI 1.24-7.81). There was an even greater breast cancer risk among women ever employed in other vegetable farming (OR = 7.33, 90% CI 1.16-46.2). One important aspect of farming in terms of endocrine disruption is that employment tends to begin earlier than other occupations. This may impart particular risks for those in pre-pubescent or pre-parity windows of vulnerability [51].

### Plastics

The plastics manufacturing jobs held by the women who participated in this study involved primarily injection molding. Injection molding and related processes take molten mixtures of resins, monomer, multiple additives, and sometimes lamination films, and form them into plastic pieces of defined dimensions and configuration. Emissions of vapors or mists from these hot processes can include plasticizers, ultraviolet-protectors, pigments, dyes, flame-retardants, un-reacted resin components and decomposition products. Further exposure comes from skin contact in handling and performing finishing tasks [52].

Many plastics have been found to release estrogenic chemicals [53]. Furthermore such additives as phthalates, and polybrominated diphenyl ethers (PBDE) have been identified as EDCs [40]. Cumulative exposure to mixtures of various estrogenic chemicals may compound the effect [54]. Some of the monomers present in the manufacturing of polymers (such as BPA, butadiene, and vinyl chloride) have been identified as mutagenic and/or carcinogenic [55]. Several monomers, additives, and related solvents, such as vinyl chloride, styrene, and acrylonitrile have been identified as mammary carcinogens in animal studies [14].

A near doubling of the risk for female breast cancer was found among plastics and rubber industry workers (SIR = 1.8; 95% CI, 1.4-2.3) [19]. Two other studies report increased breast cancer risk among rubber and plastics workers. Gardner et al. observed an OR of 1.4; 95% CI, 0.69-2.84, p=.26 after 10 years employment [56]. Ji et al. observed an OR of 2.0; 95% CI, 0.9-4.3 for those who were ever employed as plastics process machine operators [57]. Adding weight to this is a more than quadrupling of breast cancer risk found among male workers in the rubber and plastics industries (OR = 4.5; 95% CI, 0.7-28) [58]. Villeneuve et al. [22] found an increased breast cancer risk among French plastics and rubber product makers (OR = 1.8; 95% CI, 0.9-3.5). Labrèche et al. recently found an excess risk of breast cancer for occupational exposure to acrylic fibers (OR = 7.69; 95% CI, 1.5-40) and for nylon fibers (OR =1.99; 95% CI, 1.0-3.9) when exposures occurred before age thirty-six [23]. It was also reported that exposure to acrylic and rayon fibers and monoaromatic hydrocarbons doubled the risk of estrogen/progesterone positive tumours. The observation in this study of a robust association with automotive plastics manufacturing suggests that the risk factors are widespread and common in this sector. In the geographical study area, plastics production was primarily automotive. As a result, the non-automotive group was much smaller with less statistical power to detect associations. The absence of excess breast cancer incidence among non-automotive plastics workers could also be related to the types of polymers, additives and processes used in the manufacturing of automotive versus non-automotive products.

### Food canning

Food canning industry exposures could include pesticide residues and exposures specific to canning processes involving lead (historically) and coating emissions. Canning processing has been found to significantly reduce levels of residual pesticide [59] through washing, boiling, and peeling, which conceivably expose food processing workers. Some operations in this industry produce epoxy-coated cans at the food processing facility. In others, coated cans come from a supplier and are then hot washed. In either case, it is plausible that coating constituents are released into the plant atmosphere. Unlike typical consumer exposures that occur through ingestion of food packaged in epoxy-lined cans, the exposures to BPA from heated can liners experienced by canning workers occurs primarily through inhalation. The bioavailabilty of BPA that has been inhaled or absorbed dermally has been found to be eliminated at a slower rate than BPA ingested through food or drink [60]. The associations observed for canning show higher specificity with respect to receptor type and menopausal status than was observed for automotive plastics. For etiologic effects, this would be expected because the relevant exposure in canning, if polymer-related, would be more homogeneous than that across diverse plastics manufacturing activities. There is little epidemiological research on this subject. However, Ji et al. found elevated breast cancer risk for work in food canning (OR = 3.5; 95% CI, 1.2-10.1) after adjusting for reproductive history and SES [57]. Band et al. found elevated premenopausal breast cancer risk for work in food and beverage processing (OR = 3.45; 90% CI, 1.22-9.78) [21].

### Bars-gambling

There were also important findings for those employed in bars or such gambling establishments as casinos and racetracks. The strong (OR = 2.28 after 10 years) (Table 6, model 4) but statistically significant (p=0.04 with one-tailed test) excess in breast cancer among bars-gambling workers implicates second-hand smoking [6]. Environmental tobacco smoke has been identified as a major occupational health concern in the bars-gambling industries [61,62]. The time period of lagged exposures studied here largely occurred prior to restrictive smoking regulations. Increase may also be related to disruption in circadian rhythms and decreased melatonin production resulting from work at night [63].

### Metal work

The findings for metal work, which includes foundries, metal stamping, fabrication and metalworking, have important implications for a broad range of blue collar industrial operations. Although these industries expose workers to metallic fume, metalworking fluids, PAHs, solvents, and other hazards [64,65], there has been little epidemiological research on associated breast cancer risk. A weak association was found for breast cancer risk and soluble metalworking fluids [18]. Several studies have found associations between PAH exposure and breast cancer risk [4,66]. Petralia et al. [66] found elevated breast cancer risk among premenopausal women exposed to PAHs and benzene. Risk was found to be increased among young women exposed to solvents in a variety of industrial settings [67].

### Hormone receptor-type

Despite the likely presence of diverse carcinogen or EDC exposures within industrial sectors, some distinct specificity of receptor-type associations was observed. Of sectors showing elevated breast cancer, automotive plastics and the metals-related sectors would be expected to have the most diverse mix of carcinogen and EDC exposures; the canning and bars-gambling sectors would be expected to have the least diverse. Our study found statistically significant associations with canning in two receptor types (ER+/PR- and ER-), one exhibiting a statistically significant interaction with prior farm work. If the association is etiologic, this suggests that more than one mechanism may be involved. There was also a statistically significant increase in ER- tumor status among women employed in farming. Although there has been little research in this area, Danish researchers noted an association between ER- tumor status and exposure to dieldrin [68]. Whether or not the observed differences in hormone receptor status found in this study can be explained by the current level of understanding of the impact of EDC exposures on receptor status, they are indicative of the benefit of occupational investigations with more rigorous retrospective exposure assessments for investigating endocrine and other mechanistic aspects of breast carcinogenesis [69].

### Menopausal status

Finding distinct patterns for pre- versus postmenopausal breast cancer, such as increased premenopausal breast cancer among women employed in automotive plastics, adds confidence that exposure associations may be etiologic even though the exposure specificity is limited. Some of the events leading to a cancer diagnosis could occur throughout a woman’s life, i.e., both pre- and postmenopausal exposures could be involved in postmenopausal cases. Nevertheless, observing differences on menopausal status allows examination of some mechanistic hypotheses. Other studies of premenopausal breast cancer identified smoking [46] and such EDCs as benzene and PAHs [66] as risk factors.

### Limitations

Selection bias arising from participation that jointly depends on exposure history and case-status is unlikely to have played a significant role because study candidates did not know the intent of the study, and the participation rate among cases was relatively high. Among controls there would be even less likelihood of an exposure-driven decision. Uncontrolled confounding was undoubtedly present which is one reason why income terms were included in the model. Many lifestyle and health-related risk factors are associated with income. It would be quite unlikely for minor uncontrolled confounding to produce the strong specific associations observed.

Shift work was examined but did not produce statistically significant findings. In a study aggregating diverse workplaces, it is likely that exposures themselves depend on shift, making any shift-effect difficult to interpret. For example, not all employers operate a midnight shift and that shift can have maintenance, support and custodial activities that are absent in day-shift work and that could influence types or levels of exposures.

Exposure assessment based on survey instrument-derived work histories coded in NAICS and NOC categories has inevitable misclassification that dilutes or occludes observable associations [70]. Changing trends in technology and manufacturing are a further source of misclassification, possibly playing a role in some sectors like food manufacturing and dry cleaning. In the case of the food sector, focus on the specific subsector for canning produced a stronger association.

Models using the additive relative rate specification fit less well than with the loglinear choice, which assumes an exponential rather than linear dependence on the exposure metric. This suggests that the weighting scheme assuming a 10-fold higher exposure with “high” vs. “moderate” may have understated this ratio.

There was an under-representation of potentially highly exposed migrant farm and greenhouse laborers because they were not treated at the regional cancer center. This exclusion may have underestimated the risk estimate. Furthermore the role of carcinogens and EDCs that are ubiquitous in society will be underestimated in this study against the inflated background.

While this study was unable to identify exposure to specific chemicals, associations were observed between breast cancer carcinogens and EDCs. EDCs and biological windows of vulnerability are not currently considered in the establishing of occupational exposure limits (OELs). These findings, along with mounting evidence from other recent studies of harm from low level EDC exposure, point to the need to re-evaluate OELs and their relevancy in regulatory protection.

## Conclusion

A growing body of scientific evidence suggests that mammary carcinogens and/or EDCs contribute to the incidence of breast cancer [4,6,14]. Yet there remain gaps and limitations. This exploratory population-based case–control study contributes to one of the neglected areas: occupational risk factors for breast cancer. The identification of several important associations in this mixed industrial and agricultural population highlights the importance of occupational studies in identifying and quantifying environmental risk factors and illustrates the value of taking detailed occupational histories of cancer patients.

## Abbreviations

BPA: Bisphenol A; BMI: Body mass index; CI: Confidence interval; EDC: Endocrine disrupting chemical; ER: Estrogen receptor; Ind: Indicator variable; IpAg: Interaction with farming; NAICS: North American Industry Classification System; NOC: National Occupational Classification; OELs: Occupational exposure limits; OR: Odds ratio; pk-yrs: Smoking pack years; PR: Progesterone receptor; SIR: Standard incidence ratio; WRCC: Windsor Regional Cancer Center.

## Competing interests

The authors are free of financial or other conflicting interests related to the design, conduct, interpretation, or publication of the research. The findings and conclusions in this report are those of the authors and do not necessarily represent the views of the US National Institute for Occupational Safety and Health (NIOSH).

## Authors’ contributions

JB, MK, AW, AR, IL and MG contributed to the concept and design of the study. HA-Z and KS co-ordinated the informed consent and referral process from the Windsor Regional Cancer Center and the accessing of tumor hormone receptor status for each case. EM-T was responsible for the design and implementation of the study database program. JB and MK supervised data collection and, along with RD, assessed exposure values. RP constructed the analysis files, designed the statistical models and performed the analysis. RP, MK, JB, MB, AW and MG interpreted the data and contributed to the writing of the manuscript and all authors participated in revisions of the manuscript and approved it.
